# Wearable Triboelectric Nanogenerator from Waste Materials for Autonomous Information Transmission *via* Morse Code

**DOI:** 10.1021/acsami.1c20984

**Published:** 2022-01-20

**Authors:** Bhaskar Dudem, R. D. Ishara G. Dharmasena, Raheel Riaz, Venkateswaran Vivekananthan, K. G. U. Wijayantha, Paolo Lugli, Luisa Petti, S. Ravi P. Silva

**Affiliations:** † Advanced Technology Institute, Department of Electrical and Electronic Engineering, University of Surrey, Guildford, Surrey GU2 7XH, U.K.; ‡ Wolfson School of Mechanical, Electrical and Manufacturing Engineering, Loughborough University, Loughborough LE11 3TU, U.K.; § Free University of Bolzano-Bozen, Piazza Universitá 1, Bolzano-Bozen 39100, Italy

**Keywords:** triboelectric nanogenerator, waste materials, flexible electronics, wearables, autonomous internet of things

## Abstract

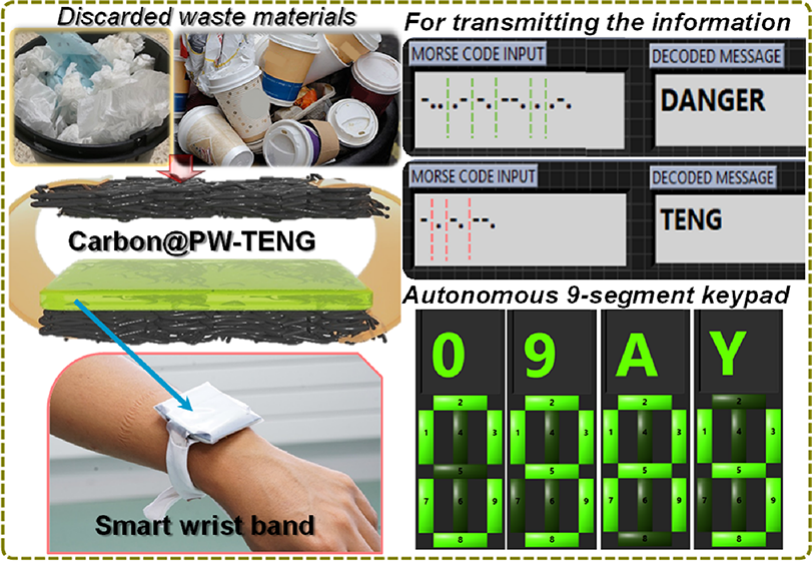

Electronic waste produced by plastic, toxic, and semiconducting components of existing electronic devices is dramatically increasing environmental pollution. To overcome these issues, the use of eco-friendly materials for designing such devices is attaining much attention. This current work presents a recycled material-based triboelectric nanogenerator (TENG) made of plastic waste and carbon-coated paper wipes (C@PWs), in which the PWs are also collected from a waste bin. The resultant C@PW-based TENG is then used for powering low-power electronic devices and, later, to generate a Morse code from a wearable for autonomous communication. In this application, the end users decode the Morse code from a customized LabVIEW program and read the transmitted signal. With further redesigning, a 9-segment keyboard is developed using nine-TENGs, connected to an Arduino controller to display the 9-segment actuation on a computer screen. Based on the above analysis, our C@PW-TENG device is expected to have an impact on future self-powered sensors and internet of things systems.

## Introduction

The gradual and worldwide deployment of 5G technology calls for contactless operations, which are anyway in great demand due to the increased use of the internet of things (IoTs) in both the consumer electronics and the industrial sectors.^1−3^ The IoT era is in urge of better, smaller, and more efficient portable and wireless technologies in appliances. To better address the IoT needs, these miniaturized electronic components need to consume less power to operate, thereby decreasing the power consumption.^4,5^ Renewable technologies such as indoor solar will develop based on the current low cost of solar modules, where the drive is to make the energy materials even cheaper.^6^ To date, there are a lot of IoT-related applications in various fields such as wearables, healthcare, traffic monitoring, agriculture, hospitality, water supply, smart grid, and so forth.^7−10^ Sensors play a major role as a primary component in the IoT systems for performing a crucial function in effectively improving the human lifestyle.^7−10^ In order to operate these sensors and systems, at least a few milliwatts of power is necessary, and this can be provided by built-in components such as triboelectric nanogenerators (TENGs).^11^ Recently, TENGs have been employed for human machine interfacing by directly converting the biomechanical energies into sensory information and have been used in gloves, touch pad, exoskeleton, personal health care, and security applications such as Morse code generators.^11−14^ Among these applications, Morse code generation and transmission has gained a huge attention due to its practical usage in telecommunication and broadcasting for security applications, as well as for SOS safety signaling for public safety and emergency. So far, there are several reports demonstrating the process of Morse code generation using TENGs made of different triboelectric layers. Nevertheless, all of these have drawbacks, such as low stability, decoding, efficiency, and so forth.^15^ The present manuscript overcomes the lag by introducing the solution of decoding the generated Morse codes from the TENG actuation from biomechanical motions employing a LabVIEW code, enabling the use of the TENG-based Morse code generator for real-time emergency applications. Also, the Morse code generator is made in a wearable wrist band-type device which can be worn and be used from remote locations. This approach paves way toward smart emergency systems with IoT technology in the near future.

Historically, within biomechanical energy scavenging, the most promising devices are TENGs, which were invented by the Wang’s group in 2012.^16^ TENGs are considered extremely suitable to power portable and wearable electronics due to their high adaptability, simple design, ease of fabrication, and cost-effectiveness.^17^ TENGs work on the principle of contact electrification and electrostatic induction to harness waste mechanical energy.^18,19^ TENGs can also be used for scavenging various sources of mechanical energy such as wind energy,^20,21^ water wave energy,^22^ and vehicle and human body motion.^23,24^ Previous research on nanogenerators provided solutions for enhancing the output power of the TENGs by inducing surface modifications,^20^ introducing charge trapping layers,^25^ and ion-induced enhancements,^26,27^ thereby increasing the practical applicability of nanogenerators.^28^ TENGs were also combined with other energy-harvesting technologies such as solar cells and electromagnetic generators, yielding hybrid nanogenerators that are able to operate in a broader operating frequency ranges (1–8 Hz).^29−32^ Besides, the piezoelectric materials were also used in combination with the triboelectric polymers as active layers to form hybrid devices with enhanced output performances.^33−35^ As a result of this performance, although the TENGs are promising to power IoT and an alternative to the conventional batteries, most of them consists of plastic and toxic materials which could again lead to producing plastic and e-wastes, as aforementioned. In this context, the use of eco-friendly and recycled or reused plastics will help develop such TENGs as a promising solution that also helps to reduce e-wastes.

The present manuscript provides a solution to this issue, by reusing the plastic waste as well as the use of carbon-coated recycle paper wipes (PWs) to fabricate the active layers of an eco-friendly TENG. The electrical response of a resultant carbon-coated PW-based TENG (C@PW-TENG) is studied extensively under various mechanical parameters. The electrical output response of the device is then validated using the distance-depended electric field (DDEF) model simulations. The experimental and simulation outputs are compared, resulting in a close match with each other, proving that the experimental output of the C@PW-TENG behaves in a predictable and consistent manner with respect to the DDEF equations. Further validating the electrical output, the device is used to power up low-power electronic components. To demonstrate the practical application of the C@PW-TENG, the device is re-designed as a wristband structure and used as a Morse code generator that generates and transmits Morse codes, which are then decoded using a LabVIEW module. In addition, the C@PW-TENGs is made into a 9-segment keypad, which is connected to an Arduino controller that can display numbers and letters can be further useful for emergency communication applications. From the device output and the real-time applications, the C@PW-TENG device proves that it can be used as an emergency communication device and has potential to be used with IoT systems.

## Results and Discussion

**Figure 1:**
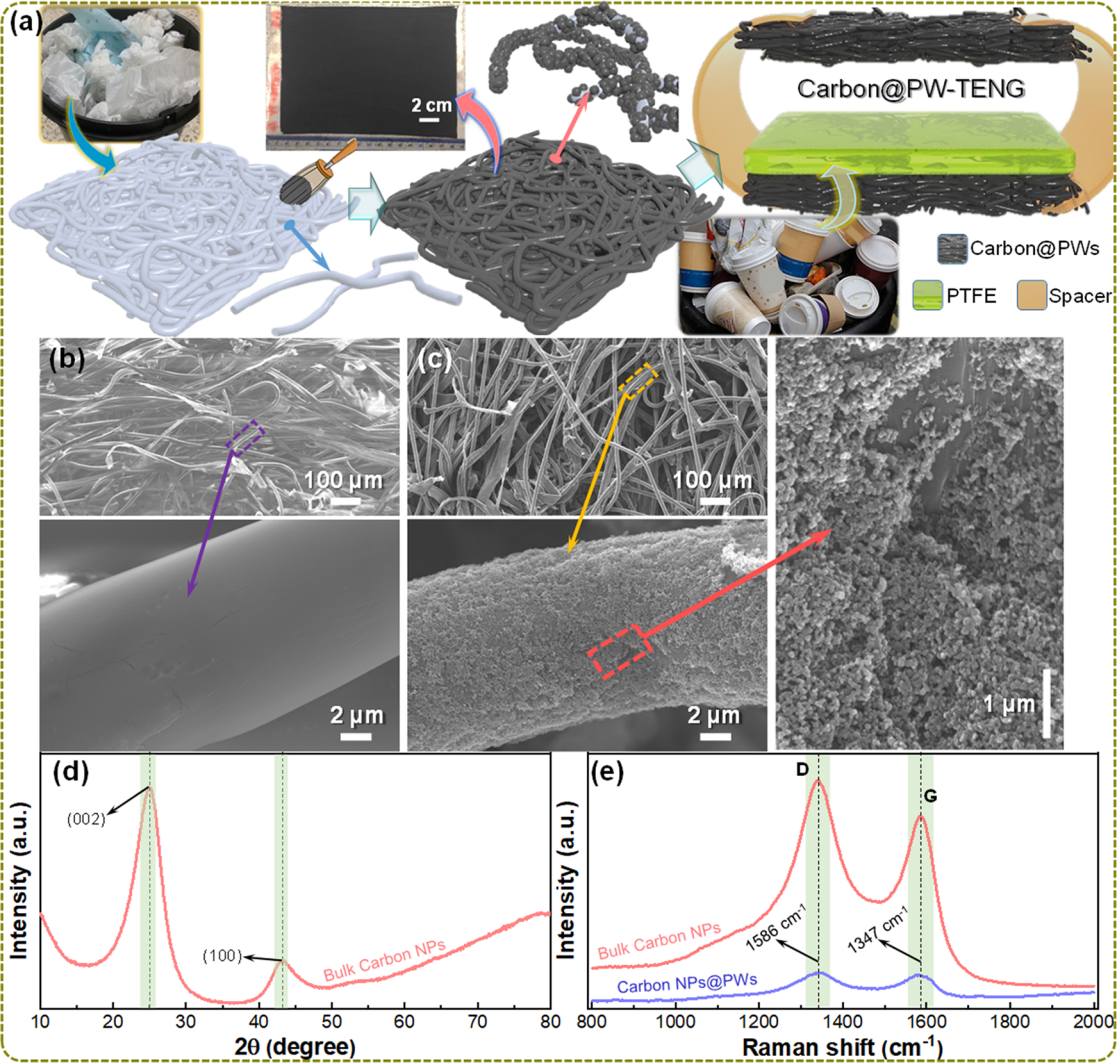
Schematic illustration and characterization of carbon-coated paper wipes (PWs). (a) Schematic diagram to illustrate the fabrication of carbon-coated discarded PWs (C@PWs), as well as the TENG realized with these PWs and discarded plastic cups. FE-SEM images of PWs at different magnifications, (b) before and (c) after coating with the carbon NPs. (d) XRD and (e) Raman spectra analysis of bulk carbon NPs and C@PWs.

A flexible and eco-friendly carbon-based TENG is developed using waste and recycled products, as illustrated in Figure 1a. First, the discarded PWs collected from the waste bin are coated with a carbon slurry by a facile, cost-effective, and high-throughput brush painting method, followed by the curing treatment. The detailed fabrication processes of carbon-coated PWs (C@PWs) is described in the Experimental Methods section. As a result, the carbon nanoparticles (NPs) are firmly coated on the micro-fibrous-networked PWs over a large area. The photographic image shown in the inset of Figure 1a clearly illustrates the carbon coated uniformly on PWs over an area of ∼200 cm^2^. To develop a flexible and inexpensive TENG device, one piece of C@PW with an area of 25 cm^2^ is used as a bottom electrode, and polytetrafluoroethylene (PTFE) collected from the waste plastic coffee cups is glued on top of it, which can function as the negative triboelectric material. Another piece of C@PW with an area is 25 cm^2^ is placed on top of it while maintaining a gap between them by utilizing flexible tape as a spacer (Figure 1a). This C@PW will function as the top electrode as well as the positive triboelectric material of the TENG. Figure 1b,c shows the scanning electron microscopy (SEM) images of PWs before and after coating with the carbon NPs. These SEM images clearly illustrate that the carbon NPs with an average diameter of 60 nm are uniformly coated across each micro-fiber of C@PW, whereas the micro-fiber of non-coated PW is almost plain without any impurities.

Furthermore, the physical, structural, and crystalline characteristics of bulk carbon NPs and C@PW are analyzed by X-ray diffraction (XRD) and Raman spectroscopy (Figure 1d,e). The XRD patterns of bulk carbon NPs (Figure 1d) exhibit two intense peaks at the 2θ values of 25 and 42.2°, which belong to the (002) and (100) crystalline plans of carbon.^36,37^ Furthermore, the XRD pattern of PWs before and after coating with the carbon were also examined (Figure S1; Supporting Information), clearly displaying similar characteristics. Moreover, the intense peaks of these PWs only belong to the cellulose, as no peaks related to carbon have been observed.^38,39^ This might be attributed to a very thin carbon layer coated on a PW, which is difficult to distinguish using the XRD. However, the Raman spectroscopy measurements are further employed to characterize the carbon-coated on PWs and bulk carbon NPs (Figure 1e). Raman spectra of bulk carbon NPs is clearly illustrating two sharp D and G bands at 1345 and 1588 cm^–1^ that are related to diamond and graphite, respectively, which match with the amorphous carbon phase, as reported previously.^36,37^ The Raman spectra of C@PW is also exhibiting both the bands and they are well-matched with the carbon. Therefore, these studies are clearly confirmed that the carbon NPs are well coated on PWs.

**Figure 2:**
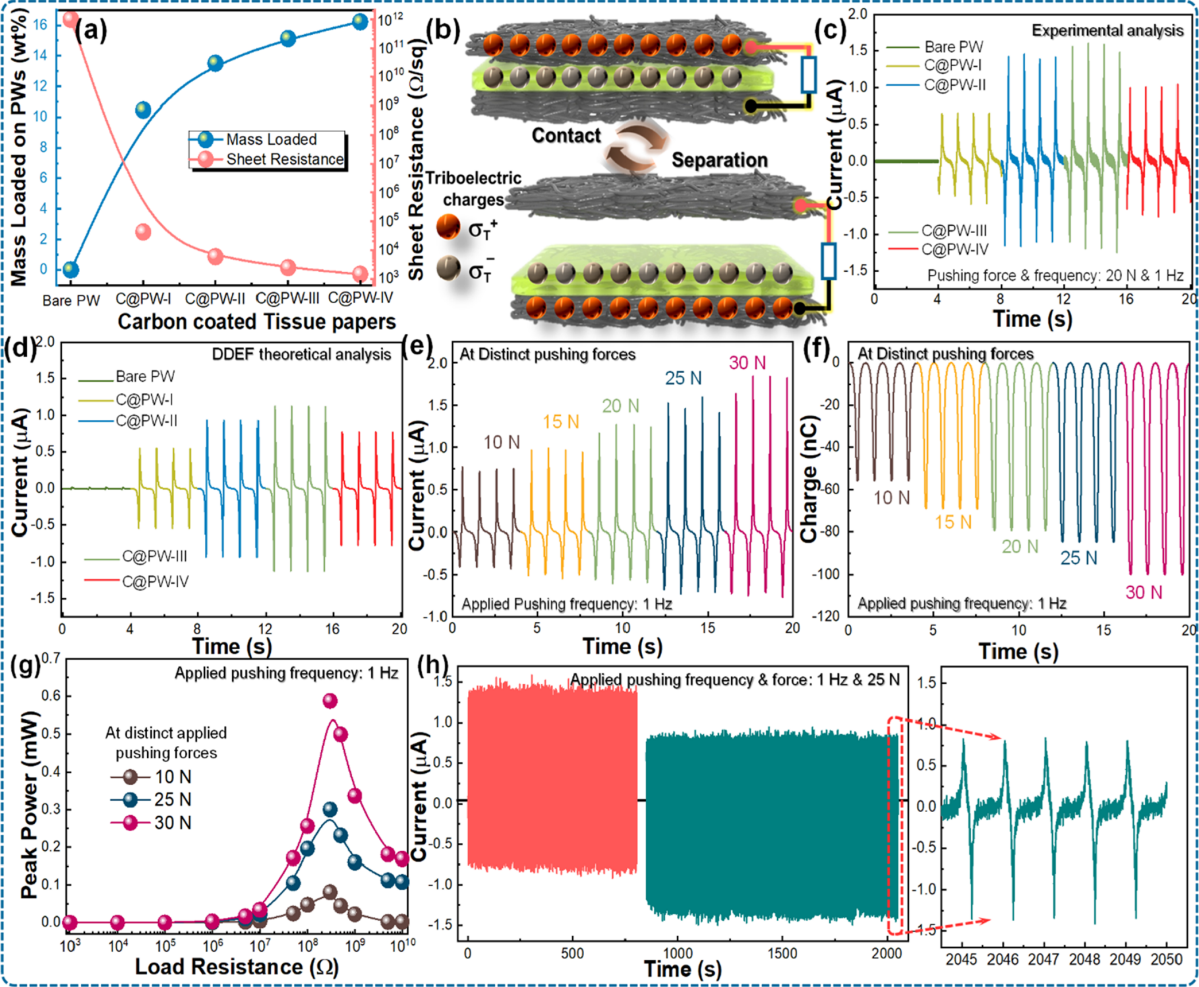
Experimental and theoretical analysis to investigate the effect of electrical performance of the C@PW-TENG device as a function of carbon mass-loaded on PWs and mechanical pushing force, its robustness behavior. (a) Weight percentage of carbon NPs loaded on different PWs and its sheet resistance. (b) Structure model and charge distribution across the individual components of the C@PW-TENG used for DDEF theoretical simulations. (c) Measured and (d) DDEF theoretical simulated electrical output *I*_SC_ curves of C@PW-TENGs as a function of carbon mass loaded on PWs. Measured electrical output (e) *I*_SC_, (f) *Q*_SC_, and (g) peak power of the C@PW-TENG at different mechanical pushing forces ranging from 10 to 30 N. (h) Robustness test of the device examined over several pushing cycles.

Herein, the various carbon mass-loaded PWs are utilized to examine their influence on the output performance of C@PW-TENGs. In order to prepare them, the carbon paint with various thicknesses is brush-coated repeatedly on PWs, as described in the Experimental Methods section. Figure 2a depicts the weight percentage (wt %) of carbon mass loaded on various PWs and their corresponding sheet resistance (*R*_S_) values. As expected, the *R*_S_ of the C@PWs is reduced by increasing the wt % of carbon mass loaded onto the PWs and eventually reaches saturation (Figure 2a). Furthermore, the influence of the wt % of carbon mass loaded on PWs on the electrical output performance of corresponding C@PW-TENGs is examined by the experimental and theoretical simulations (Figure 2c,d). The working mechanism of the C@PW-TENG to convert the mechanical energy into the electricity is schematically illustrated (Figures 2b and S2), explained as well (see Discussion-S1; Supporting Information). Figures 2c,d and S3 shows the measured electrical output including the short-circuit charge (*Q*_SC_), short-circuit current (*I*_SC_), and open-circuit voltage (*V*_OC_) curves of the corresponding C@PW-TENGs for different carbon mass-loaded PWs, while the pushing force and frequency are maintained at 25 N and 1 Hz. As the carbon mass-loaded onto the PWs is increased from 0 to 15.13 wt %, the *Q*_SC_, *I*_SC_, and *V*_OC_ values of C@PW-TENGs are gradually enhanced reaching maximum values of ∼1.56 μA, 78 nC, and 80 V, respectively. This is attributed to two factors. First, as the carbon loading increases, the quantity of carbon NPs on the positive triboelectric contact surfaces increases. This increases the effective surface area which is in triboelectric contact with negative (PTFE) triboelectric surface, resulting in an increased surface charge density. The use of nanomaterials to obtain such performance improvements is in fact a common practice for TENG designs, as reported in previous studies.^21,40,41^ Second, as evident from Figure 2a, increasing the carbon loading or the thickness of the carbon layer can significantly increase the conductivity of both the electrodes of the C@PW-TENG. Better conductivity helps in efficiently transferring the charges between the electrode of TENG, which results thereby in a higher electrical output. However, by further increasing the carbon mass loaded onto the PW to 16.23 wt %, even though the *R*_S_ of the corresponding C@PW is reduced, the electrical output of the respective TENG is reduced instead of increasing. This is against the typical trend expected for the TENG outputs, which can be attributed to the change in the surface characteristics of the triboelectric contact layers. To evaluate this hypothesis, the triboelectric surface of the PTFE layers opposite to bare PW, C@PW-II, C@PW-III, and C@PW-IV samples were analyzed using SEM and elemental mapping (Figure S4). This analysis indicates that there is minimal transfer of carbon NP on the PTFE surface corresponding to PW (Figure S4a) and C@PW-II (Figure S4b) surfaces, whereas a small amount of carbon NPs can be observed on the PTFE surface related to C@PW-III (Figure S4c). On the other hand, the PTFE surface related to the C@PW-IV (Figure S4d) shows significant quantities of carbon NP transfer, almost covering the entire PTFE surface. This suggests that, at low carbon loads, there is no or minimum carbon transfer to the PTFE surface which does not obstruct the triboelectric charge generation process. However, as the carbon loading is increased in C@PW-IV, large amounts of carbon NPs are transferred to the PTFE surface causing significant surface contamination. Therefore, the triboelectric contact effectively happens between two surfaces coated with the same material, which causes a significant drop in their surface charge density as well as the output performance of TENG. This hypothesis aligns well with the extensive theoretical and experimental work presented recently on the materials transfer characteristics between triboelectric contact surfaces.^42,43^ Overall, increasing the carbon NP loading increases the TENG outputs up to a threshold and eventually reduces afterward.

Furthermore, the DDEF model was used to assess the charge density and simulate the output generation characteristics of the TENG structures (see Discussion-S2; Supporting Information). As shown in Figure S3a, the DDEF simulations provided a close match to the experimental *Q*_SC_ outputs. However, the simulated *V*_OC_ outputs are observed to be largely different compared to the measured values (Figure S3b) that is usually observed due to the very high internal impedance of TENGs, which influence the measured output voltage in its open-circuit configuration. Besides, a similar match for the *I*_SC_ outputs can be observed (Figure 2c,d) at the same approximated charge density values. The DDEF model simulations, therefore, confirm that the charge density of the triboelectric surfaces initially increases with the carbon loading (from bare PW to C@PW-III showing an increase from 0.4 to 35 μC/m^2^) and shows a subsequent reduction (24 μC/m^2^) in the case of C@PW-IV. This aligns well with the hypothesis developed in the previous paragraph regarding the output behavior of the TENG devices. The electrical response analysis of the C@PW-TENG under multiple mechanical stimuli was also investigated, as shown in Figure 2e,f. The electrical output such as the peak-to-peak *I*_SC_ and *Q*_SC_ values of the C@PW-TENG increases from 1 to 2.5 μA and 58 to 100 nC, with the increase in applied force from 10 to 30 N. This is due to the change in internal resistance of the C@PW-TENG with the elastic deformation of the PTFE layer. Similarly, under the applied stress, there could be a chance for small shrinkage in the C@PWs layer. Further, the impedance matching analysis is performed under different forces (10–30 N), with different load resistance (*R*_L_) values, as shown in Figure 2g. Upon increasing these loads, the power of device also increases, which is due to the increase in the electrical current. The peak/maximum power (*P*_max_) of the C@PW-TENG increases from 0.1 to 0.58 mW upon increasing the loads from 10 to 30 N. Further, the stability of the fabricated device under robust condition was analyzed under a pushing force and frequency of 25 N and 1 Hz, over 2000 s (Figure 2h). In addition to the stability, a switching polarity test is also conducted to confirm that the electrical output is solely generated from the triboelectric effect and not by other parameters, such as instrumental or human errors influencing its output. Hence, the initial 750 s were measured under forward connection (polarity) of the electrometer probes and the remaining period was at reverse connection (the inset shows the peak pattern observed during the reverse connection). The stability test analysis proves that the device is capable of harvesting energy over a long period, delivering the electrical output steadily without fluctuation. Besides, by switching the polarity, an almost 180° phase shift is noticed in the electrical output current of C@PW-TENG, which clarifies that the electrical output is strictly generated from the C@PW-TENG owing to the triboelectric effect. Furthermore, the durability (or endurance) of the proposed device produced even after a month was examined by measuring its electrical output up to 18 days (daily measured 10,000 cycles), as shown in Figure S5. These studies clearly illustrated no significant change in an average output current of the C@PW-TENG over an almost 18 days, supporting that its output performance is relatively stable for several cyclic compressions and days, showing its potential for practical applications. For further validating the practical applicability of C@PW-TENG, they were employed to power low-power portable electronic systems such as LEDs and LCDs *via* a commercial capacitor (Figure S6). These experiments clearly demonstrate that the proposed C@PW-TENG is adequate to significantly be employed for self-powered applications.

**Figure 3:**
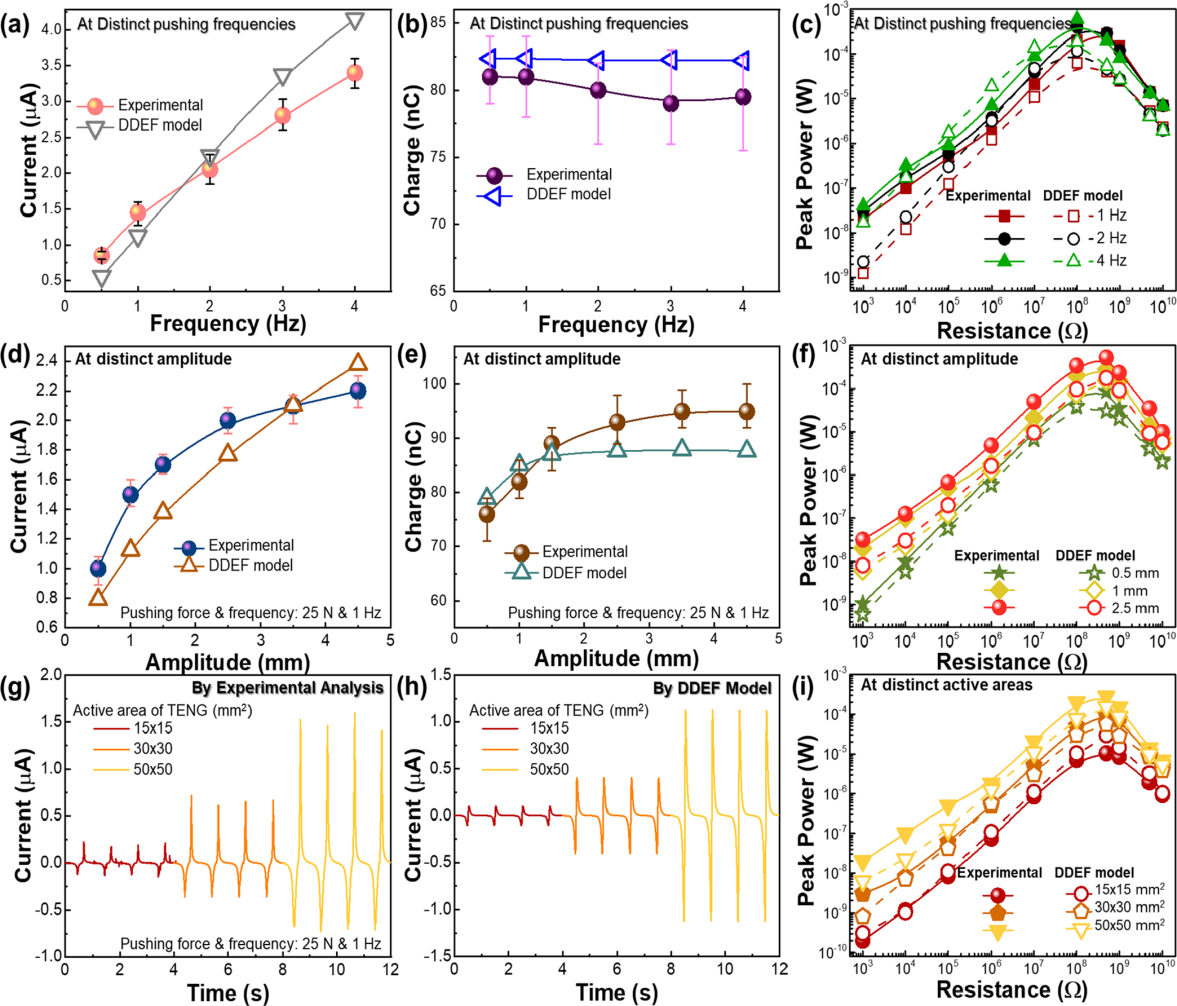
Electrical output response of C@PW-TENGs as a function of frequency, amplitude, and active area. Comparison of experimentally measured peak *I*_SC_ and *Q*_SC_ values of the C@PW-TENG against the DDEF theoretical simulation results, under an increasing (a,b) mechanical pushing frequency from 0.5 to 4 Hz, and (d,e) amplitude ranging from 0.5 to 4.5 mm; error bars specify the standard deviation of 50 readings. (g) Measured and (h) DDEF theoretical simulation electrical output *I*_SC_ curves of the C@PW-TENG, against increasing the surface area of active materials. Peak output power values of the C@PW-TENG noticed by the experimental and DDEF simulation studies against distinct (c) mechanical pushing frequencies, (f) amplitudes, and (i) surface areas.

In addition to the robustness, the ability of the TENG to respond under various frequencies, amplitudes, and device active areas is also important to investigate and validate with the theoretical outcomes, for significantly employing it for distinct practical/commercial applications. So, the electrical response of the C@PW-TENG under various frequencies ranging from 0.5 to 4 Hz were evaluated by experimental and DDEF model simulations (Figure 3a–c). As per the experimental outcomes, the increasing frequency significantly increases the output current (Figure 3a), whereas the charge remains almost constant (Figure 3b). The DDEF model investigations also agree with this trend. Since the maximum amplitude of the TENG movement is kept constant in this case, a constant number of charges are exchanged between the electrodes of the TENG during its movement. However, as the frequency is increased, the rate of the movement of charges increases, resulting in an increase in the current output. Furthermore, the *P*_max_ of the C@PW-TENG device was calculated upon applying different oscillating frequencies, and the output response is compared with the DDEF model (Figure 3c). Due to relatively higher current outputs at increasing frequencies, the output power increases, where an increase from 1 to 4 Hz results in a *P*_max_ increment from 0.2 to 0.61 mW through a *R*_L_ of 100 MΩ in the experimental scenario (Figure 3c). The DDEF model simulation results show a similar trend where the *P*_max_ output is recorded at 4 Hz frequency, through a *R*_L_ of 100 MΩ. Further, the electrical output performance of the C@PW-TENG was studied under various amplitudes of movement (Figure 3d–f). By varying the amplitude from 0.5 to 4.5 mm with the constant pushing force and frequency (at 25 N and 1 Hz), the current increases from 1 to 2.2 μA (Figure 3d) and the transferred charge increases from 75 to 95 nC (Figure 3e), respectively. The amplitude with larger displacement leads to a larger separation of the friction layer, which creates a larger potential difference between the electrodes, enabling the device to generate high electrical outputs. The electrical outputs are again validated by performing the DDEF simulations, which indicated a close match with the experimental outcomes (Figure 3d,e). It should be noted that at higher amplitude movements, there is a slight mismatch of the current output trend between the experimental outputs and DDEF model simulations (Figure 3d). This is due to the sampling rate limitations of the electrometer during experimental measurements, especially at higher amplitude movements of the TENG. Extending the electrical analysis, we calculated the *P*_max_ of the device at three different amplitudes (0.5, 1, and 2.5 mm) under various *R*_L_’s. The *P*_max_ output increases from 86 μW for 0.5 mm to 0.52 mW for 2.5 mm through a *R*_L_ of 500 MΩ (Figure 3f). The DDEF model simulations indicated a relatively similar trend (Figure 3f). Furthermore, the electrical response was cross-verified by fabricating three devices with different triboelectric contact surface areas (Figures 3g,h and S7). The electrical response was measured with the pushing force, frequency, and amplitude of 25 N, 1 Hz, and 1 mm. The devices generated an electrical output current of 0.3 μA (15 × 15 mm^2^), 1 μA (30 × 30 mm^2^), and 3.1 μA (50 × 50 mm^2^) that clearly shows the 2-fold to 4-fold increment in the electrical response (peak-to-peak) compared with the 15 × 15 mm^2^ device (Figure 3g). With the increase in active area of the device, the charge transfers between the triboelectric layer’s increases, which results in a higher potential difference between the electrodes, creating higher electrical outputs. The electrical output with respect to scaling up the active area was also validated through the DDEF model simulations, which indicate a relatively similar trend to experimental analysis (Figure 3h). Figure 3i shows the impedance matching analysis and *P*_max_ calculation for scaled devices with different active areas. The 50 × 50 mm^2^ device shows the *P*_max_ of 0.28 mW at a *R*_L_ of 500 MΩ, whereas the 15 × 15 mm^2^ device exhibited the lowest power of 10.46 μW. This is attributed to its higher electrical output from the large area device. From the above studies (Figure 3), it can be concluded that the experimental outcomes are well validated with the DDEF simulations, indicating a similar trend as the experimental scenario.

**Figure 4:**
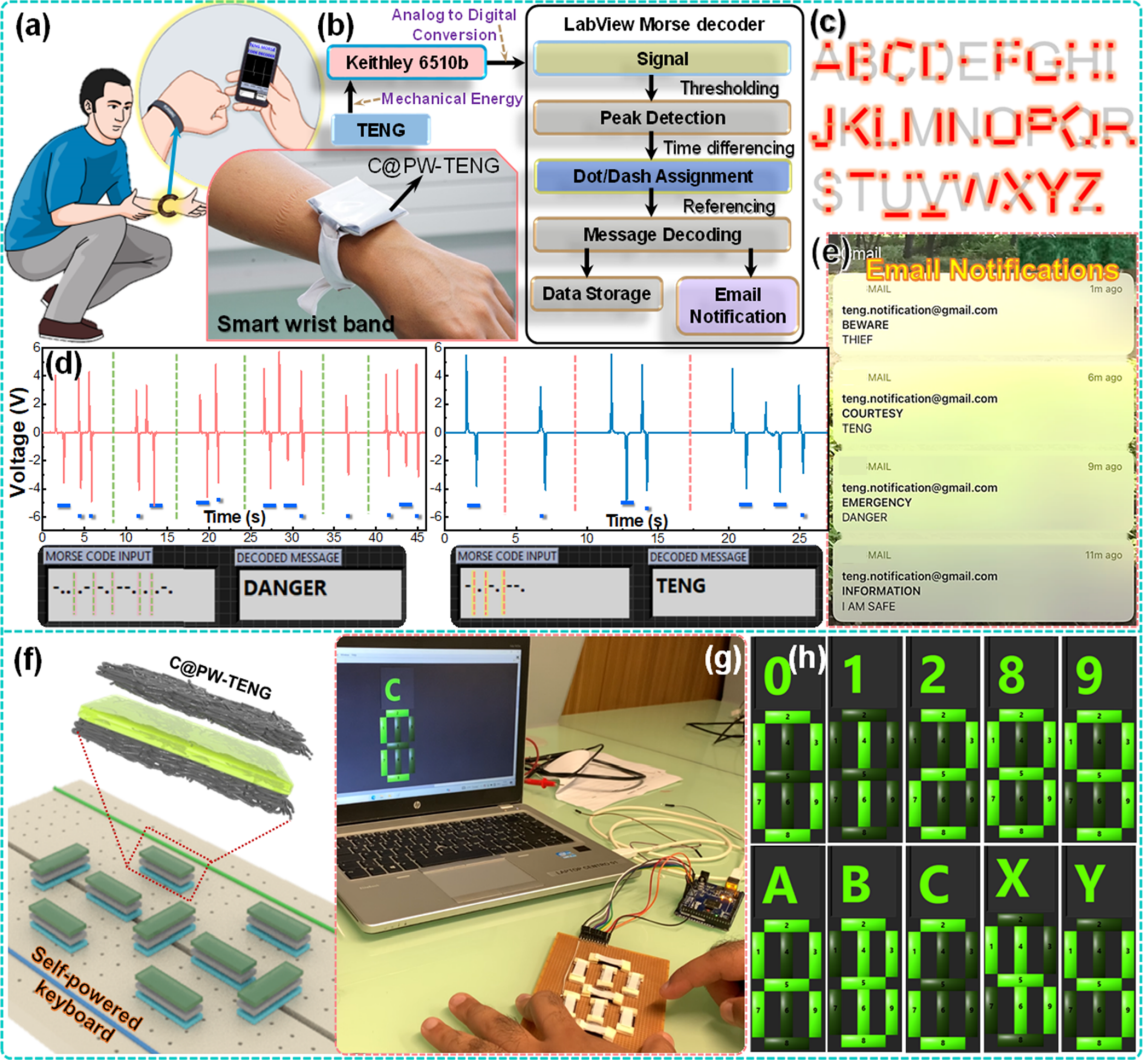
Self-powered wearable and information transformation applicability function of the C@PW-TENG. (a) Schematic and photographic illustration of the C@PW-TENG as a wearable intelligent wristband to transfer the information under emergency *via* the Morse code. (b) Process flow chart to identify the Morse code signals followed by the message decoding and sending a notification through the LabVIEW platform, to alert the take career of individuals in case of emergency. (c) Representation for the International Morse code for alphabets. (d) Electrical output response noticed by tapping the smart wristband with finger according to a Morse code, denoting DANGER and TENG, respectively. (e) Email notification delivered to the take career of the individual *via* the LabVIEW Morse decoder. (f) Schematic illustration and (g) photographic image of the self-powered 9-segment keyboard developed by the C@PW-TENG. (h) Numbers and alphabets displayed by touching the C@PW-TENG-based 9-segment keyboard *via* the Arduino circuit board.

To extend the usage of the C@PW-TENG devices for real-time application, we have re-modeled the C@PW-TENG as a smart wristband device as shown in Figure 4a. Figure S8 illustrate the digital photographic images of smart wristband developed by utilizing the C@PW-TENG device with an active area of 30 × 30 mm^2^. As shown in Figure S8, prior to use it as a wristband, the C@PW-TENG was well packed into the polythene to protect it from the moisture. Typically, the moisture in the external environment also strongly influences the output performance of TENGs. As per our previous works,^24,28^ the effect of moisture on the output performance of TENGs can be minimized by the proper packing process. Thus, we believe such polythene wrapped around the C@PW-TENG can be efficient enough to sustain in the humid environment and produces a stable electrical output. For further confirmation, the electrical output performance of the packed C@PW-TENG was measured under various humidity conditions (Figure S9) and noticed an almost identical electrical output even though the moisture in the environment increased. Such a well-packed C@PW-TENG was further wrapped or inbuilt into the textile; since a major part of the resultant device is composed of textile fabric, it is easy for the device to be used as a wristband. As discussed before, the advancement in electronics and miniaturization of components paves way toward wearable electronics and IoTs, in which information, communication, and security are of great interest. Among the information and communication devices, the Morse code transmitter plays a major role in security applications. Herein, the wearable C@PW-TENG device is used as a Morse code generator by tapping the device in a Morse code generation pattern of dot and dash by touch and release for dot and touch-hold-release for dash. The corresponding peak pattern and response for dot and dash is shown in Figure S10. The schematic of Morse code decoding is shown in Figure 4b with mechanical energy harnessing and converting it to a digital signal and decoding it using a LabVIEW module. With the operation of device as the Morse code generator under dot, dash pattern, the English alphabets from A to Z are generated (Figure 4c), and the message “DANGER” and “TENG” are transmitted and decoded, as shown in Figure 4d. The decoder screen made by the LabVIEW graphical user interface is shown in Figure S11. Similarly, we have generated a Morse code signal for transmitting the messages “THIEF” and “I AM SAFE”, which are shown in Figure S12. The decoded information is then programmed to be stored in a storage system and the message can then be sent to email, as shown in Figure 4e. The advantage of using the C@PW-TENG device as a Morse code generator is that it can work as long as it can generate the signal and there is no requirement of any power source for the signal generator. Extending the application of the C@PW-TENG device, a 9-segment keypad was developed using nine C@PW-TENG devices and connected to the computer screen using the Arduino controller, which is shown in Figure 4f,g. The keypad is capable of providing input and can be attached on doors as security locks. The number and alphabets from 0 to 9 and A to Z are displayed by tapping the keypads, as shown in Figure 4h. As a proof of concept, the device actuation and the corresponding numbers were displayed in the computer screen. This has the potential to be used for self-powered data transmission and for a door lock security system that is wireless in connection with IoTs. The experiment and the real-time applications prove that the C@PW-TENG device is a promising candidate to be a power source for low-power electronic devices, as well as to be used in real-time information and communication systems.

## Conclusions

In summary, a TENG consisting of waste product C@PWs and PTFE was fabricated using simple and easy fabrication techniques, which can be used as a wristband-type IoT device. The device fabrication was entirely based on the concept of waste recycling by collecting the waste PWs and coffee cup lids. As an energy-harvesting unit, it generates a maximum electrical output current of 3.5 μA and power of 0.61 mW, under an applied pushing frequency and force of 4 Hz and 25 N. The C@PW-TENG is then made into a wristband-type device to be used as a wearable device for performing real-time applications of a Morse code generator and a self-powered 9-segment keyboard. The Morse code signal for numbers and letters from the C@PW-TENG was demonstrated by the successful transfer and decoding with the help of a LabVIEW module. Similarly, a 9-segment display was operated by actuating the right devices to display the numbers on the computer screen with the help of an Arduino board for security and user identification applications. The above concepts and demonstrations prove that the C@PW-TENGs can be used extensively for various IoT-based applications that can improve the quality and lifestyle of society.

## Experimental Methods

### Materials

The chemicals and reagents including Super P conductive carbon black (MTI Corporation), polyvinylidene fluoride (PVDF; Sigma-Aldrich), and *N*-methyl-2-pyrrolidone (NMP; Sigma-Aldrich) are directly used without any further purification.

### Coating of the Carbon NPs on the PWs

Initially, the discarded PWs collected from the waste bins were cleaned and further coated with the carbon NPs through a brush painting method. Herein, the carbon painting slurry is prepared by grinding the 95 wt % of Super P conductive carbon black and 5 wt % of PVDF binder in NMP solution. The well-grinded carbon slurry was brush-painted onto the PWs at a speed of about 2 cm/s and then dried at 90 °C overnight in an oven. In this study, the PWs with the different weight percentages of the carbon loaded onto it are utilized, which are developed by painting the several layers of carbon (or by painting the carbon repeatedly just after drying) onto the PWs. Eventually, these C@PWs are cut into the desired dimensions and utilized to develop flexible and inexpensive TENGs.

### Design of the Flexible and Cost-Effective TENG

The above-mentioned C@PWs are employed as the electrodes as well as the positive triboelectric material of TENGs. Besides, the PTFE collected from the waste plastic coffee cups are employed as a negative triboelectric material of the TENG. Herein, a PTFE film with a thickness of ∼50 μm is realized by cutting off the discarded plastic coffee cup into the desired dimension followed by the hot-pressing treatment. Such a PTFE film is further glued onto the C@PW and used as one component, and the only C@PW was used as another component of the TENG by separating with a certain gap.

### Characterization

The FE-SEM (JEOL JSM-7100F), XRD (PANalytical X’Pert Pro), and Raman spectroscopy (XploRA PLUS Confocal Raman Microscope) measurements were used to examine the surface morphology, structural information, and crystalline characteristics of carbon NPs and carbon-coated PWs. The sheet resistance of C@PWs was measured by the two-point probe multimeter at 10 different areas (with the surface area of 1 cm^2^), and an averaged value is noted. An electrometer (Keithley 6514) was used to measure the electrical outputs including the *V*_OC_, *I*_SC_, and *Q*_SC_ curves of the C@PW-TENG. A customized bespoke linear motor setup was used to apply the compressive mechanical forces with multiple magnitudes and cyclic frequencies onto the C@PW-TENG.

## Supporting Information Available

XRD pattern of PWs with and without carbon, schematic for the working mechanism of C@PW-TENG, electrical output of the TENG with different C@PWs, SEM images of PTFE opposite to various C@PWs after several pushing cycles, theoretical simulation explanations, endurance test of the TENG, commercial application demonstrations, electrical output of TENGs with different active areas, photographic images of the wrist-band-like C@PW-TENG, electrical output response of the smart wrist band with different messages, and LabVIEW graphical user interface used for the Morse decoder (PDF)
