# Supplementary material for: Wearable Triboelectric Nanogenerator from Waste Materials for Autonomous Information Transmission via Morse Code
Source: ACS Appl Mater Interfaces. 2022 Jan 20;14(4):5328–37. doi: 10.1021/acsami.1c20984 (PMC9007404; doi:10.1021/acsami.1c20984)
Supplement: Supplementary file 1 — am1c20984_si_001.pdf [file am1c20984_si_001.pdf]

# Supporting Information

## Wearable Triboelectric Nanogenerator from Waste Materials for Autonomous Information Transmission *via* Morse Code

*Bhaskar Dudem,[a] R. D. Ishara G. Dharmasena,[b] Raheel Riaz,[c] Venkateswaran Vivekananthan,[a] K.G.U. Wijayantha,[b] Paolo Lugli,[c] Luisa Petti,[c] and S. Ravi P. Silva,[a*]*

[a]Advanced Technology Institute, Department of Electrical and Electronic Engineering, University of Surrey, Guildford, Surrey, GU2 7XH, United Kingdom.

[b]Wolfson School of Mechanical, Electrical and Manufacturing Engineering, Loughborough University, Loughborough, LE11 3TU, United Kingdom.

[c]Free University of Bolzano-Bozen, Piazza Universitá 1, 39100, Bolzano-Bozen, Italy.

*Corresponding author Email: s.silva@surrey.ac.uk (S. R. P. Silva)

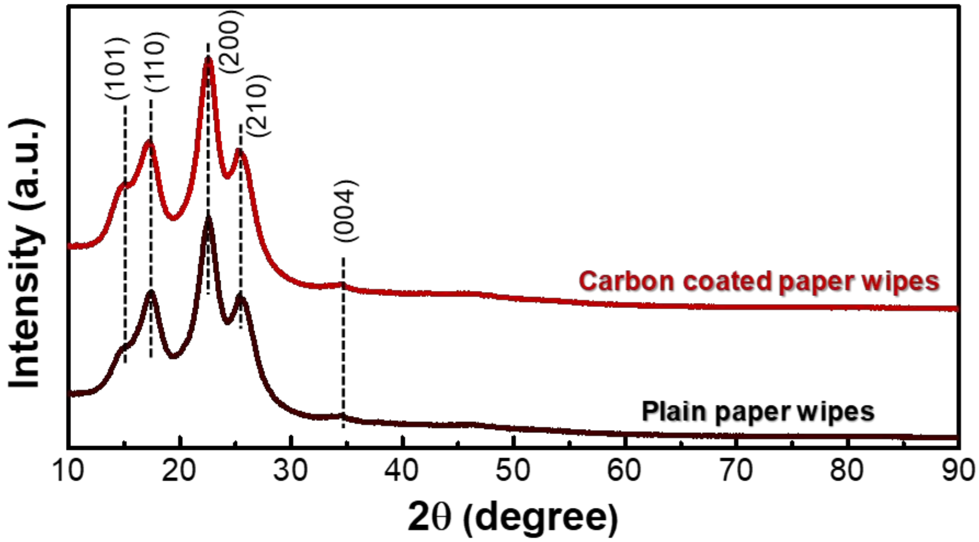


**Figure S1.** XRD pattern of plain and carbon coated paper wipes, which are displaying the similar characteristics. This might be attributed to a very thin carbon layer coated on a paper wipe, which is difficult to identify by the XRD.

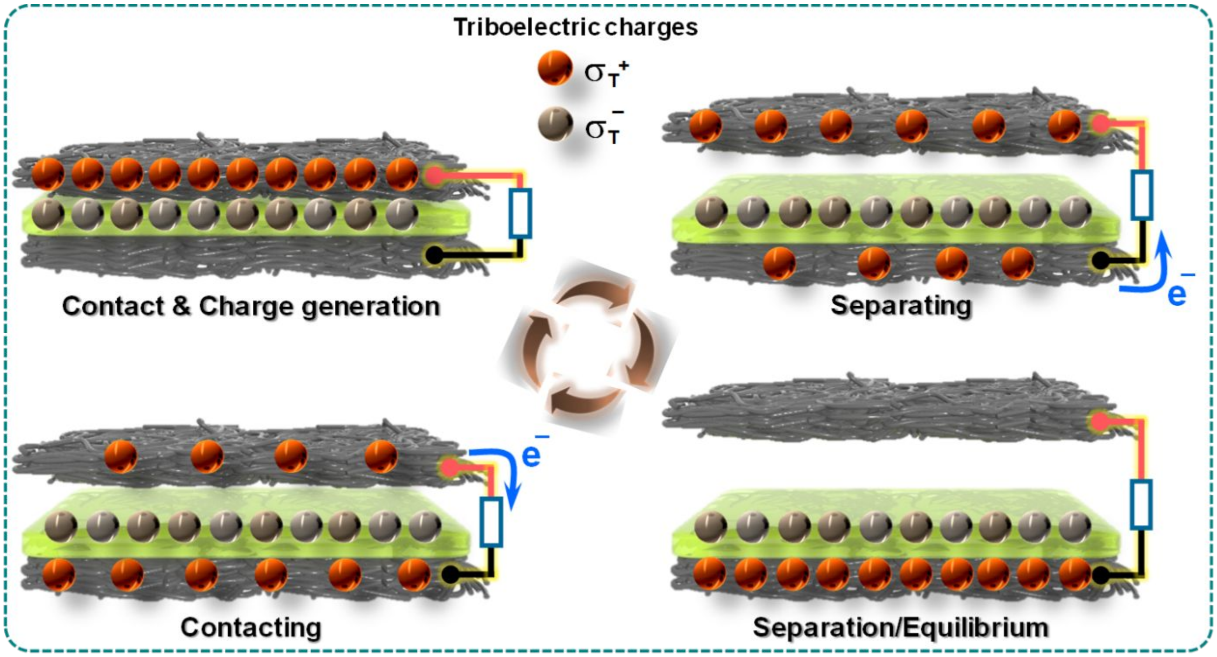


**Figure S2.** Schematic diagram to illustrate the working mechanism of carbon coated paper wipes based TENG (C@PW-TENG).

**<u>Discussion-S1:</u>** *Working Mechanism of C@PW-TENG*

As shown in **Figure S2**, the charging of the surface is initiated when the PTFE and C@PW are bought in to contact. However, these charges with opposite polarity and equal in number are on the same plane, so no output is produced. When PTFE and C@PW start to separate, these initiated charges (or electrons) start to flow between the respective electrodes (i.e., across the bottom & top C@PWs), which result in the electric current flow from top to the bottom electrode. This occurs owing to the adjustment of charged species bound to atoms and molecules of dielectrics, known as "polarization". However, this charge flow between electrode will continue until it reaches electrostatic equilibrium. As the PTFE and C@PW are bought closer again, the polarization decreases, and the charges start flow back again and creating a current flow from bottom to the top C@PW electrodes.

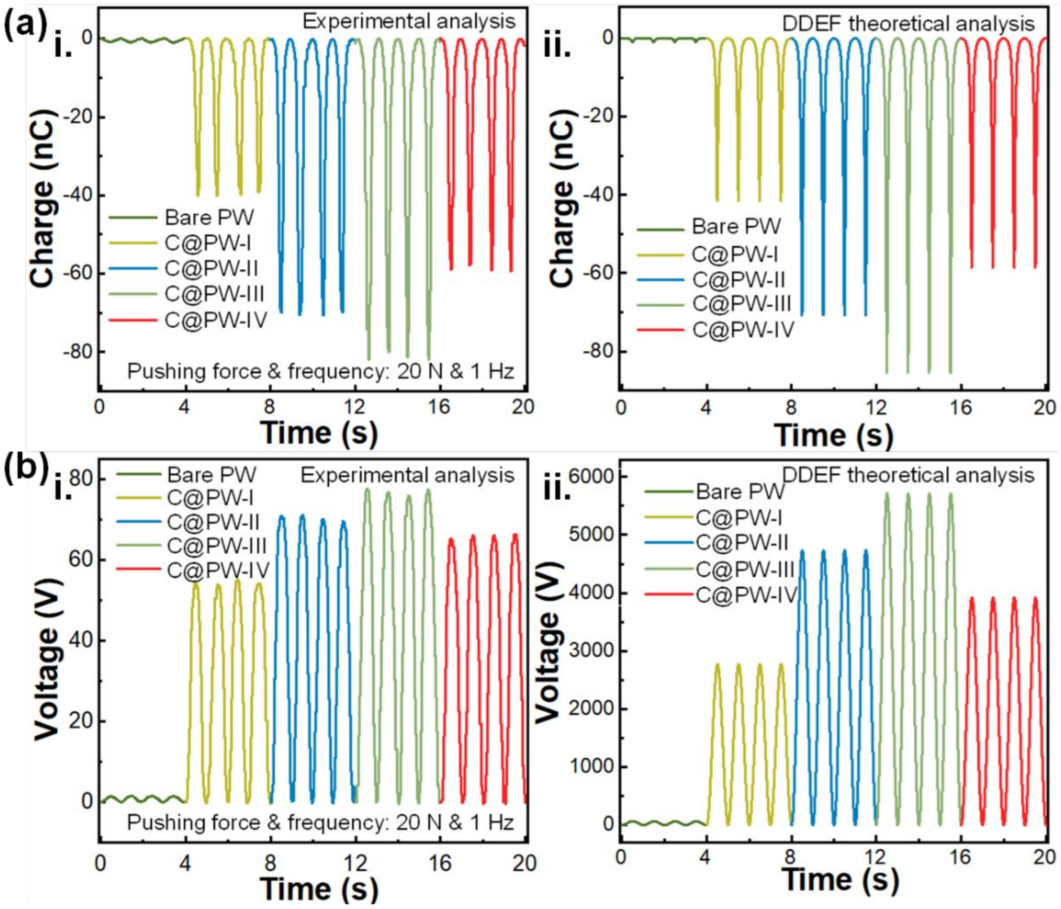


**Figure S3.** Compression of (i) measured and (ii) DDEF theoretical simulated electrical output (a) $Q_{SC}$ and (b) $V_{OC}$ curves of C@PW-TENGs as a function of carbon mass loaded on PWs.

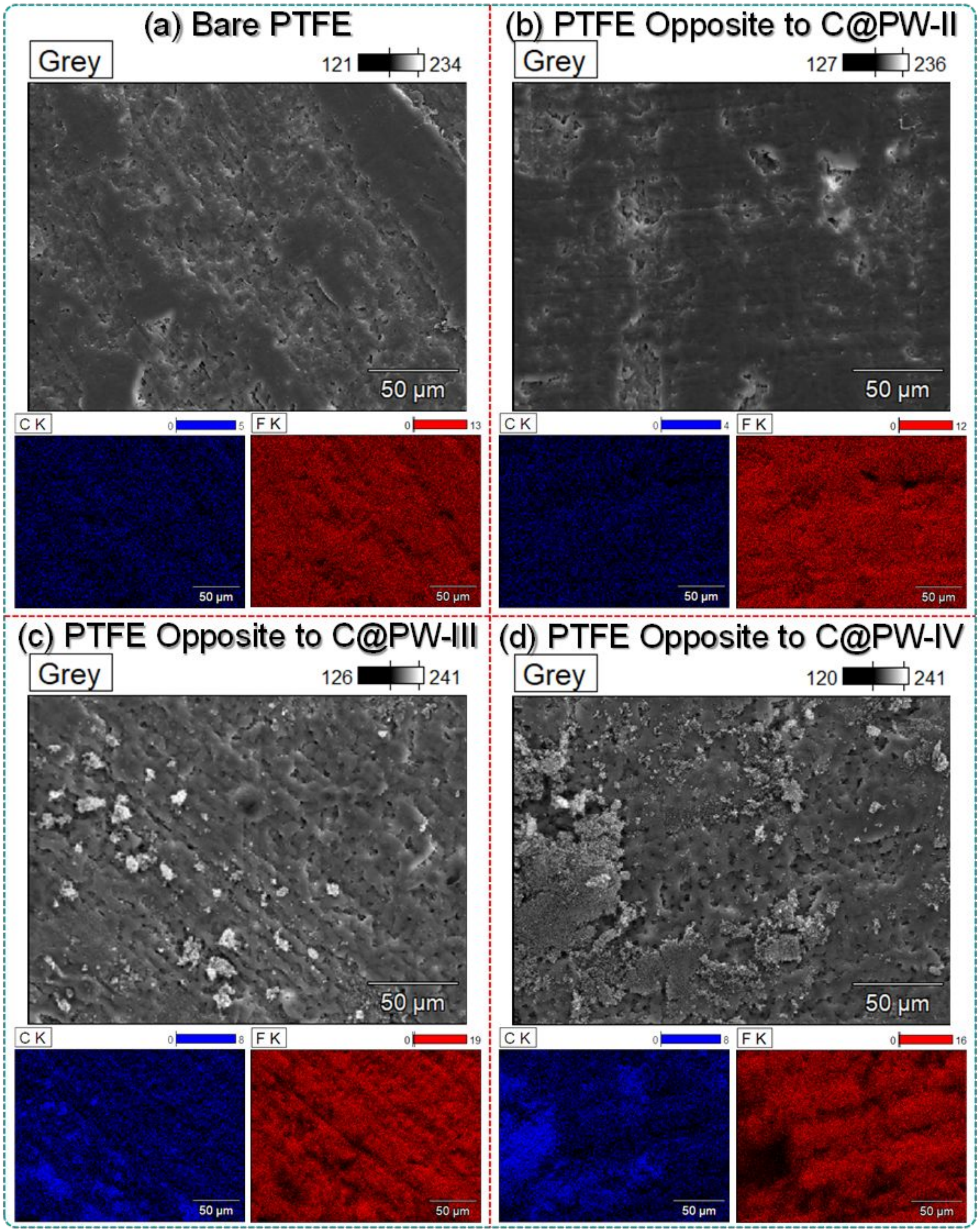


**Figure S4.** The SEM and elemental mapping images of triboelectric surface of the PTFE layers opposite to bare PW, C@PW-II, C@PW-III, and C@PW-IV samples.

**Discussion-S2:** *Theoretical simulations.*

The distance-dependent electric field (DDEF) model was used to simulate the outputs of the TENG devices using techniques similar to that used in our previous work.[i-iii] The relationship between the primary TENG parameters (which include the length and width of triboelectric layers (L, W), triboelectric layer thickness ($x_1$, $x_2$), separation between the TENG layers (z(t)), permittivity (ε), and triboelectric charge density ($\sigma_T$)) and the electric field of a charged TENG surface is given by the DDEF equation:

$$E_z = \frac{\sigma_T}{\pi\varepsilon}\arctan\left(\frac{L/W}{2\left(z/W\right)\sqrt{4\left(z/W\right)^2 + \left(L/W\right)^2 + 1}}\right) = \frac{\sigma}{\pi\varepsilon_0}f(z) \qquad (1)$$

By applying eqn. 1 to each of the triboelectric surfaces and the electrode interfaces, the current, charge, voltage, and power output of the TENG can be evaluated. For the theoretical simulations in this paper, the DDEF model modified for the single electrode mode contact-separation TENG was used. Similar to our previous studies,[i-iii] TENG parameters of each scenario corresponding to the size, layer thickness, dielectric constant, motion profile were used in the DDEF model to first theoretical evaluate the charge density of the triboelectric surfaces based on experimental $Q_{SC}$ and $I_{SC}$ values (apart from the device parameters described in the experimental section, the dielectric constant of 2 was used for the PTFE layer). Once the charge density was approximated, the $Q_{SC}$, $I_{SC}$, $V_{OC}$ and Power outputs were simulated using the DDEF model equations, as shown in Fig2-3 in the main text and Fig. S3 & S6 in the supporting information. For all these theoretical simulations, the charge density of each scenario was approximated as 0.4 μC/m$^2$ for the bare PW, 17 μC/m$^2$ for C@PW-I, 29 μC/m$^2$ for C@PW-II, 35 μC/m$^2$ for C@PW-III, and 24 μC/m$^2$ for C@PW-IV, respectively, based on the experimental measurements shown in Figure S3(C).

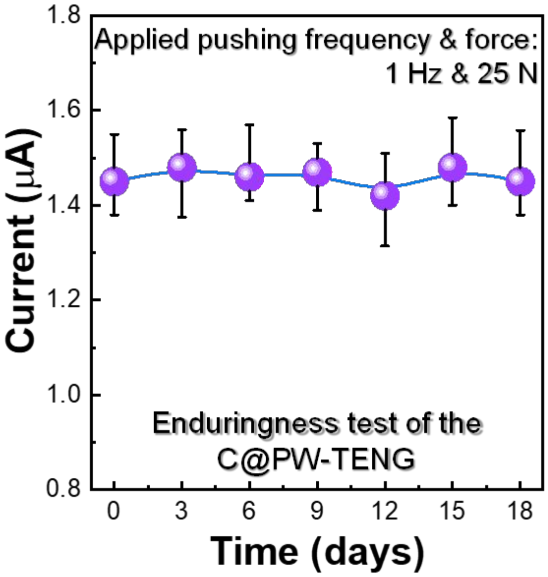


**Figure S5.** Enduringness test of the C@PW-TENG device even up to 18 days, under an applied pushing force and frequency of 25 N and 1 Hz, respectively.

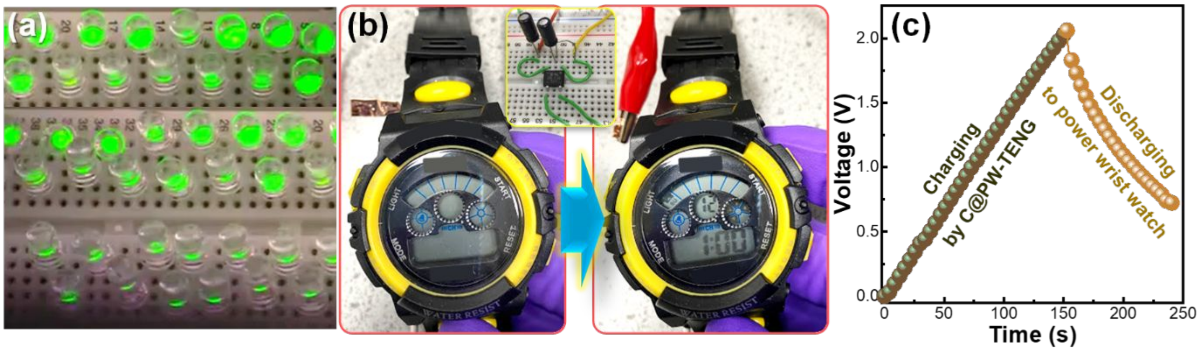


**Figure S6.** (a) Commercial LEDs connected in series and (b) wristwatch display powered either directly by the C@PW-TENG or through a rectifier followed by a capacitor. (c) The charging and discharging curve of the commercial capacitor connected in between the C@PW-TENG and wristwatch through a rectifier.

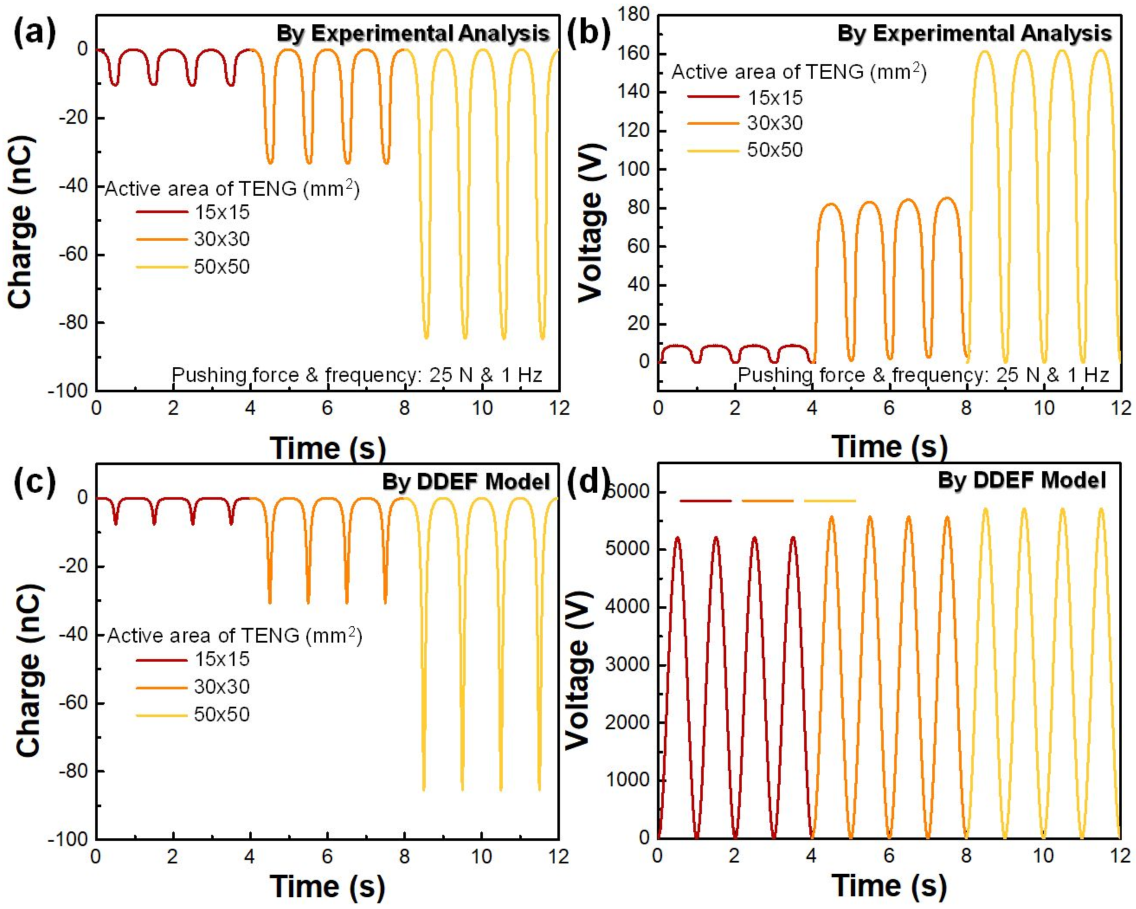


**Figure S7.** (a-b) Measured and (c-d) DDEF theoretical simulation electrical output response curves of C@PW-TENG, against increasing the surface area of active materials. The measured and theoretically analyzed electrical output response such as charge and voltage response of the corresponding devices are also well matched with the above discussion (In Figure S6)

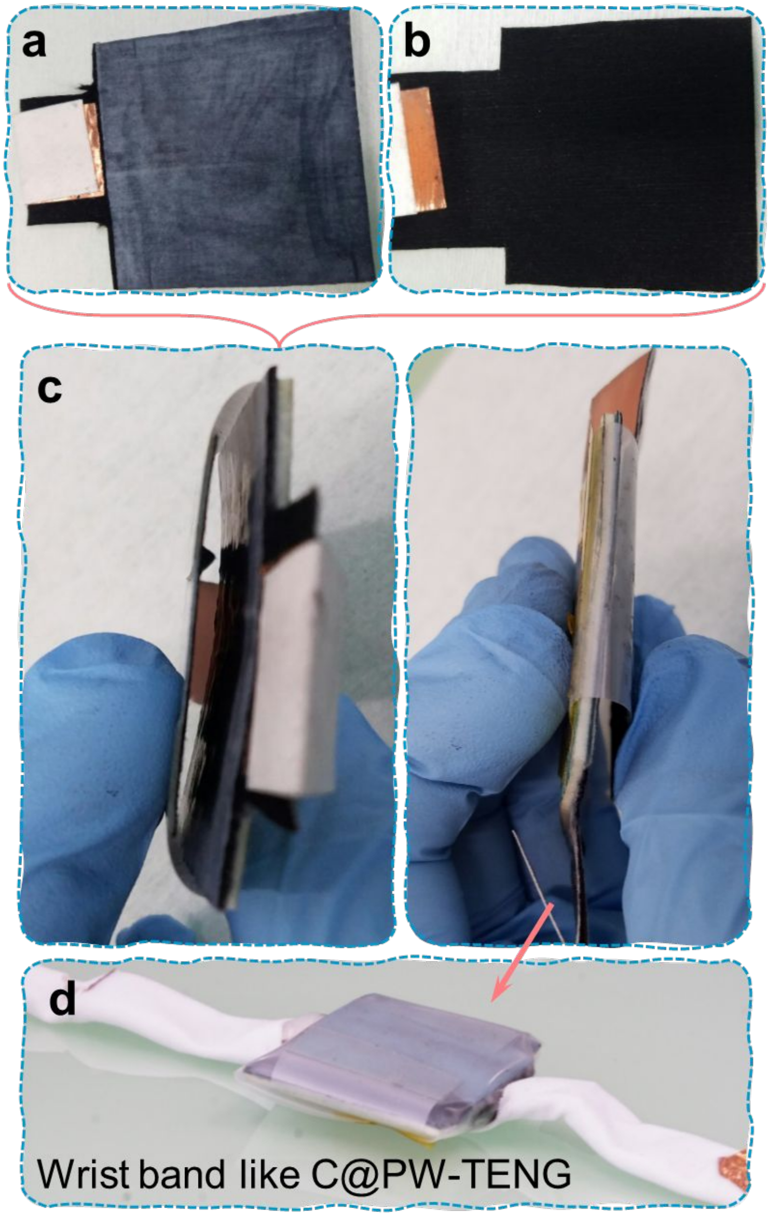


**Figure S8.** Photographic images to illustrate the assembly of different components of C@PW-TENG to design the smart wristband. (a) Bottom and (b) top components of C@PW-TENG such as PTFE/C@PW and C@PW, respectively.

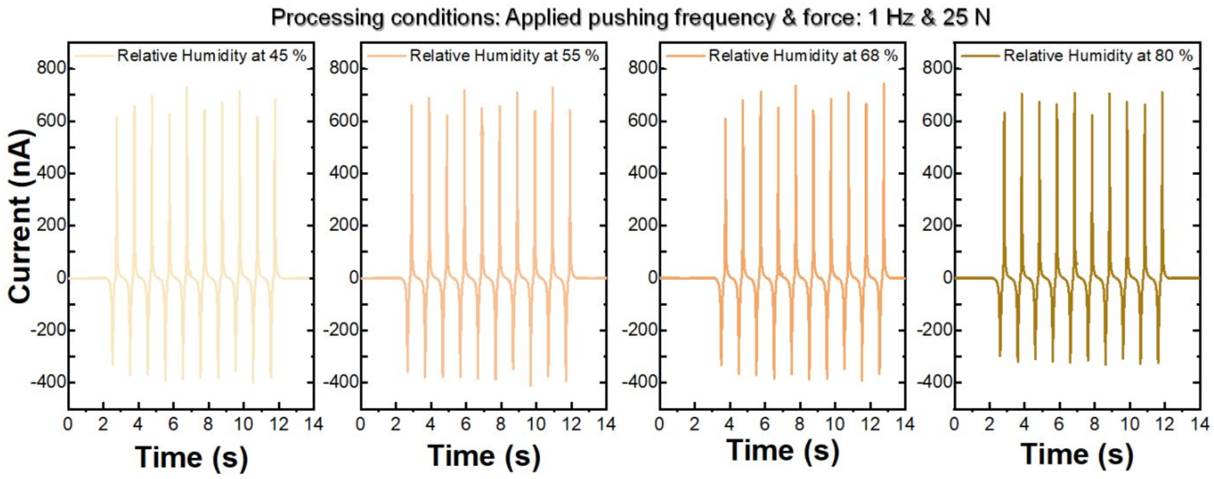


**Figure S9.** Electrical output current of the packed C@PW-TENG (with an active area of 30×30 mm$^2$) at various relative humidity conditions ranging from 45-80 %.

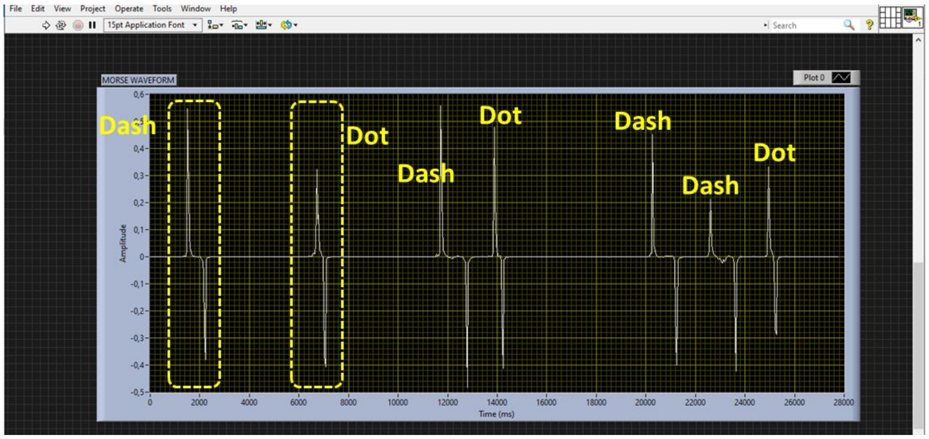


**Figure S10.** Photographic image illustrates the difference between the electrical output response of smart wrist band used to denote the "***dash***" and "***dot***" signals of Morse code. Herein, the time difference < 500 ms is maintained between the positive and negative peak to transmits ***dot* ' . '**. Whereas, the time difference > 500 ms and < 2 s is maintained between the positive and negative peak to transmits ***dash* ' - '**.

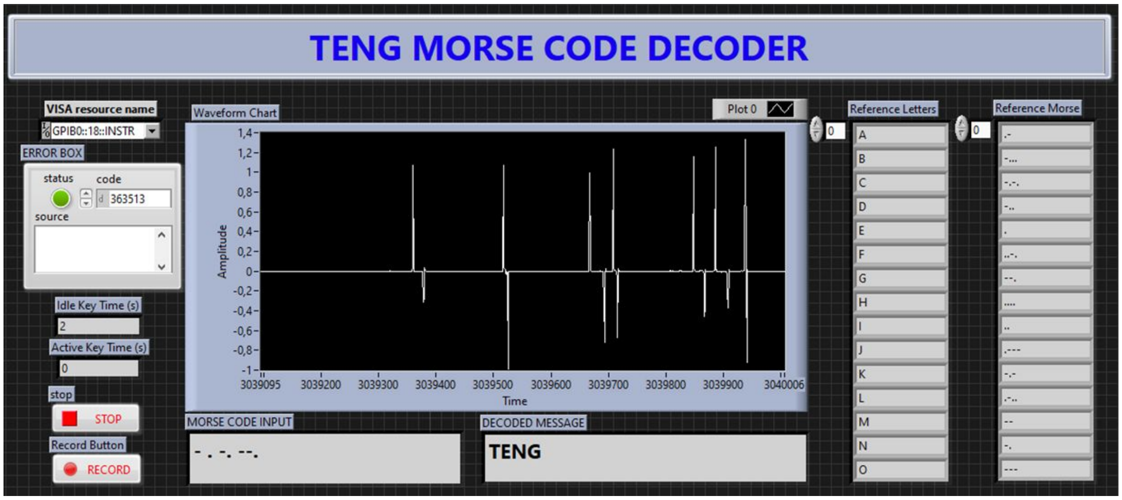


**Figure S11.** Photographic image illustrate the LabVIEW Graphical User Interface (GUI) used for Morse decoder.

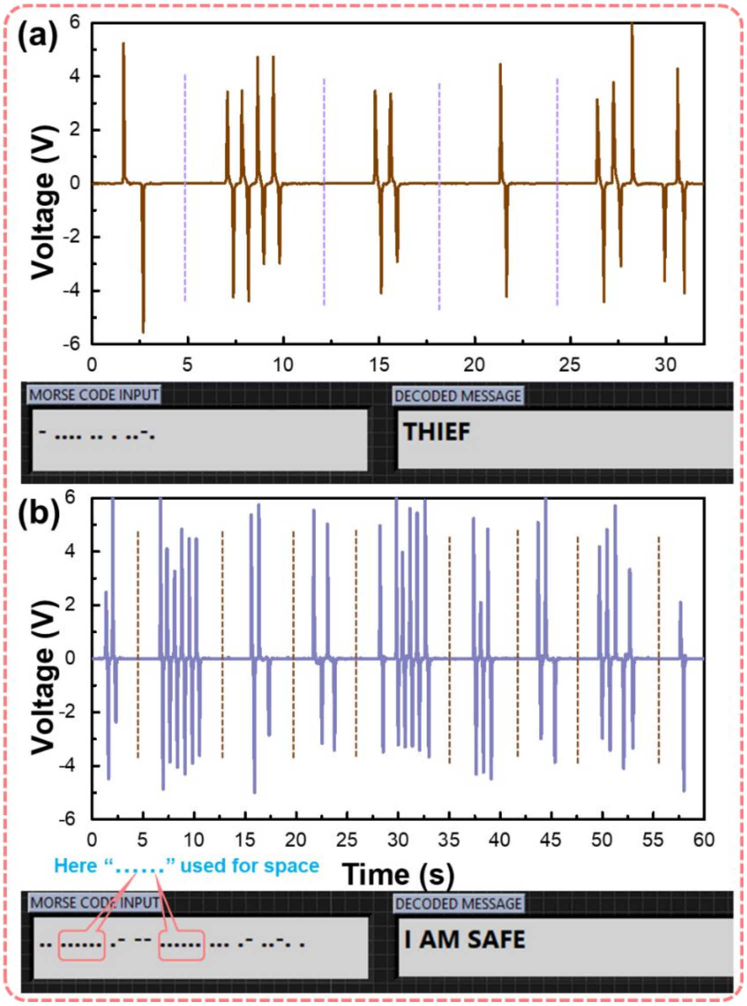


**Figure S12.** Electrical output response noticed by tapping the smart wrist band with finger according to a Morse code, denotes (a) THIEF and (b) I AM SAFE, respectively.

**References for Supporting Information:**

i. Dharmasena, R. D. I. G.; Jayawardena, K. D. G. I.; Mills, C. A.; Deane, J. H. B.; Anguita, J. V.; Dorey, R. A.; Silva, S. R. P. Triboelectric Nanogenerators: Providing a Fundamental Framework. *Energy Environ. Sci.* **2017,** *10*, 1801-1811.

ii. Dharmasena, R. D. I. G.; Deane, J. H. B.; Silva, S. R. P., Nature of Power Generation and Output Optimization Criteria for Triboelectric Nanogenerators. *Adv. Energy Mater.* **2018**, *8*, 1802190.

iii. Dharmasena, R. D. I. G.; Jayawardena, K. D. G. I.; Dorey, R. A.; Silva, S. R. P., A Unified Theoretical Model for Triboelectric Nanogenerators. *Nano Energy* **2018**, *48*, 391-400.
